# GenoMine: a CRISPR-Cas9-based kill switch for biocontainment of *Pseudomonas putida*

**DOI:** 10.3389/fbioe.2024.1426107

**Published:** 2024-09-16

**Authors:** Enrique Asin-Garcia, Maria Martin-Pascual, Claudia de Buck, Max Allewijn, Alexandra Müller, Vitor A. P. Martins dos Santos

**Affiliations:** ^1^ Laboratory of Systems and Synthetic Biology, Wageningen University & Research, Wageningen, Netherlands; ^2^ Bioprocess Engineering Group, Wageningen University & Research, Wageningen, Netherlands; ^3^ LifeGlimmer GmbH, Berlin, Germany

**Keywords:** CRISPR-Cas9-mediated biocontainment, kill switch, riboregulator, genetic circuit, *P. putida*

## Abstract

Synthetic genetic circuits have revolutionised our capacity to control cell viability by conferring microorganisms with programmable functionalities to limit survival to specific environmental conditions. Here, we present the GenoMine safeguard, a CRISPR-Cas9-based kill switch for the biotechnological workhorse *Pseudomonas putida* that employs repetitive genomic elements as cleavage targets to unleash a highly genotoxic response. To regulate the system’s activation, we tested various circuit-based mechanisms including the digitalised version of an inducible expression system that operates at the transcriptional level and different options of post-transcriptional riboregulators. All of them were applied not only to directly control Cas9 and its lethal effects, but also to modulate the expression of two of its inhibitors: the AcrIIA4 anti-CRISPR protein and the transcriptional repressor TetR. Either upon direct induction of the endonuclease or under non-induced conditions of its inhibitors, the presence of Cas9 suppressed cell survival which could be exploited beyond biocontainment in situations where further CRISPR genome editing is undesirable.

## 1 Introduction

In recent years, *P. putida* KT2440 has come to occupy a prominent place on the list of synthetic biology chassis (de Lorenzo et al., 2021; Martínez-García and De Lorenzo, 2024). Its unique metabolic and physiological properties that provide high levels of solvent tolerance and stress resistance made it first appealing for degradation studies and harsh biotransformations (Nikel et al., 2016). However, the expanding plethora of molecular and *in silico* tools for targeted genetic manipulations is behind the seemingly unstoppable rise of this bacterium as one of the preferred flexible and engineerable hosts for general metabolic engineering and bioproduction (Nikel and de Lorenzo, 2018; Martin-Pascual et al., 2021). While the toolbox of methods and genetic parts grows day by day, many of them remain unapplied and overshadowed by traditionally reliable techniques and other established elements. Specifically, numerous native and heterologous inducible expression systems (Calero et al., 2016; Martínez-García and de Lorenzo, 2017; Batianis et al., 2020) alongside different synthetic libraries of genetic regulatory elements like promoters, ribosome binding sites (RBSs) and terminators have been characterised in *Pseudomonas putida* (Zobel et al., 2015; Elmore et al., 2017; Amarelle et al., 2019; Aparicio et al., 2020; Damalas et al., 2020). However, and by the same token, the majority of these has never been exploited in an application for gene expression modulation yet, which is fundamental for a proper control of engineered metabolic pathways. At the same time, more complex elements such as riboswitches and riboregulators to fine-tune gene expression are scarce in this bacterium hindering the development of synthetic genetic circuits to tightly control cellular functions (Martin-Pascual et al., 2021).

Programming microorganisms through circuit-oriented innovations has led to novel applications and functionalities that range from diagnostics and therapeutics (Riglar and Silver, 2018) to advanced bioproduction (Xu et al., 2014; Lo et al., 2016). Moreover, genetic circuits offer excellent means for controlling cell viability and consequently for biological containment. These engineered cells would sense the environment and respond accordingly by being functional or inactivated, thus maintaining their performance only under permissive conditions (Chan et al., 2016). Specifically, kill switches are circuit systems that can result in cell death by activating lethal genes under certain conditions (Stirling et al., 2017). Addressing challenges such as high degrees of efficiency and robustness becomes imperative when building such circuits in order to eliminate all the targeted population. Additionally, the kill switch’s response needs to be quick in order to prevent instabilities and other detrimental consequences of a delayed action (Xia et al., 2019). Popular among the lethal genes used in kill switches, we encounter toxins (Chan et al., 2016; Piraner et al., 2017; Stirling et al., 2017), proteases (Chan et al., 2016) and CRISPR nucleases, such as Cas9 (Bikard and Barrangou, 2017; Rottinghaus et al., 2022). The endonuclease Cas9 creates double strand DNA breaks (DSB) in the bacterial chromosome triggering cell death (Anders et al., 2014; Jinek et al., 2014); an effect that has been reported in a variety of species. However, some bacterial cells can survive such DNA damage since some positions in the genome are targeted more efficiently than others (Cui and Bikard, 2016), a limitation that could be overcome by targeting more than one locus simultaneously. To avoid the design and utilisation of different CRISPR independent targets, the genotoxicity of Cas9 could be improved by targeting repetitive regions of the genome (Castanon et al., 2020).

Repetitive extragenic palindromic (REP) elements are highly conserved inverted repeats found in the genomes of some bacterial species (Stern et al., 1984), including *P. putida* KT2440 (Aranda-Olmedo et al., 2002). This strain has >900 REPs with a highly conserved 35 bp sequence typically occurring as single units or pairs. REPs are also the target for some insertion sequences (IS) which are small, mobile genetic elements carrying information for their own transposition (Siguier et al., 2015). Particularly, the transposase ISPpu9 is encoded seven times also in repetitive extragenic palindromic regions of the *P. putida* KT2440 genome (Gómez-García et al., 2021). Therefore, by using only the REP and ISPpu9 sequences, hundreds of parallel DSBs could be generated by Cas9 as long as those sequences were positioned next to the required protospacer adjacent motif (PAM).

With the aim of developing a quick and effective CRISPR-Cas9-based kill switch, we took advantage of the minimal PAM (5′-NNG-3′) of the ScCas9 variant (Chatterjee et al., 2018). This allowed the engineering of *P. putida* by installing into its genome a CRISPR cassette containing only two spacers to target the repetitive REP and ISPpu9 regions. To control the lethal cleavage of the strain’s chromosome, we subjected Cas9 to the control of robust genetic circuits that had previously shown outstanding performance in bacteria. These circuits include AND gates such as conventional riboregulators (Isaacs et al., 2004; Gallagher et al., 2015) and toehold riboregulators (Green et al., 2014), and an ON/OFF digitaliser module (a YES gate) (Calles et al., 2019). The basic design principle of a kill switch, however, should be a NOT, a NOR or a NAND gate to activate the lethal genes when the external signal(s) commonly administered under permissive and controlled conditions stop(s) being perceived. Consequently, the Cas9 controlled by the circuits was replaced by two repressing elements, AcrIIA4 and TetR, whereas an independent Cas9 was expressed on a different module. Circuits were used first to control the expression of the anti-CRISPR protein AcrIIA4 that inhibits the enzymatic activity of type II Cas9 proteins (Dong et al., 2017; Yang and Patel, 2017); and secondly, to control the expression of TetR, a transcriptional repressor of the pLtetO promoter (Saenger et al., 2000) which was, in turn, accommodated to control the independent Cas9.

## 2 Materials and methods

### 2.1 Bacterial strains and media

All bacterial strains utilised in this study with their respective characteristics can be found in Supplementary Table S1. Standard cultivation was done at the strains’ optimal temperature, 37°C for *Escherichia coli* and 30°C for *P. putida*, in Lysogenic Broth (LB) (10 g/L NaCl, 10 g/L tryptone, and 5 g/L yeast extract) supplemented with appropriate antibiotics according to the following concentrations: kanamycin, 50 mg/L; gentamicin, 10 mg/L; chloramphenicol, 20 mg/L. When required, relevant inducers were as well supplemented into the medium: 3-methylbenzoate at 1 mM and rhamnose at 3.75 mM, if not stated otherwise. *Escherichia coli* cells were made chemically competent as previously described (Green and Rogers, 2013) and used for cloning purposes.

Electrocompetent *P. putida* strains were prepared following previously described standard protocols (Choi et al., 2006) and used for cleavage assays and circuit experiments. Specifically, single colonies were used to inoculate 10 mL overnight cultures. The day of the transformation, 10 mL fresh cultures were reinoculated from the overnight ones and grown at 30°C and 200 rpm to an OD_600_ = 1 ± 0.2, which corresponds to 3.0 × 10^8^ CFU/mL. Cells were made electrocompetent by consecutive washing steps of 10, 2, and 1 mL of sucrose 300 mM. The washed cultures were finally resuspended in 200 μL of sucrose 300 mM and aliquoted into samples of 100 μL. Each sample was mixed with 100 ng of DNA plasmid and transferred to pre-chilled 2 mm gap electroporation cuvettes. A single exponential decay pulse was applied using the Gene Pulser X-Cell (Bio-Rad) set at 2,500 V, 200 Ω and 25 μF. Immediately after the electrical pulse, cells were resuspended in 900 μL of SOC medium (MP Biomedicals), and incubated at 30°C and 500 rpm, for 60 min. Specific plating conditions are described in the corresponding experiments.

### 2.2 Plasmids

Plasmids used in the present study are fully described in Supplementary Table S2. Primers to amplify the genetic parts (Supplementary Table S5) and to verify the products can be found in more detail in Supplementary Table S3. All genetic parts were amplified by PCR using the NEB Q5^®^ High-Fidelity DNA polymerase, according to the company’s instruction. PCR fragments were subjected to a 1% (w/v) agarose gel electrophoresis and isolated using Nucleospin Gel and PCR Clean-up (BIOKÉ) kit. Plasmids were constructed using SEVA-based backbones (https://seva-plasmids.com), and Golden Gate and Golden Gate-based SevaBrick Assembly methods (Damalas et al., 2020), unless otherwise stated, and transformed by heat-shock in chemically competent *E. coli* DH5α cells. Isolation of the plasmids was done with the GeneJET Plasmid Miniprep Kit^®^ (Thermo Scientific) and colony PCR was performed to verify the right assembly of the different fragments. Plasmid sequence was confirmed by Sanger sequencing from Macrogen (MACROGEN Inc. DNA Sequencing Service; Amsterdam, Netherlands).

First, we generated a CRISPR-Cas9 vector pSEVAb62-ScCas9-tracrRNA-crRNA_REP_ISPpu9 for the construction of the appropriate GenoMine circuit’s CRISPR array. Spacers REP and ISPpu9 (Supplementary Table S4) were introduced in pSEVAb62-ScCas9-crRNA_eforRed using the previously described protocol called “One-step Golden Gate-based cloning for the assembly of single and multiple spacers into the crRNA cassette” (Batianis et al., 2020) by replacing the eforRed chromoprotein. Once the array was built, it was PCR amplified with primers G3-G4 and cloned into a G1-G2 amplified pGNW backbone to knock it in the PP_5322 locus of the *P. putida* KT2440 genome. Plasmids pSEVAb62-ScCas9-tracrRNA and pSEVAb62-dScCas9-tracrRNA were also built without a crRNA for those experiments in which the crRNA was already installed on the genome. dScCas9 contained a D10A point mutation into ScCas9’s HNH domain and a H840A point mutation into its RuvC domain (Supplementary Table S5).

Plasmids containing the reporter *gfp* were used to evaluate how the different circuits control gene expression in *P. putida.* Plasmids pZE21 Y12 a12C GFP containing conventional riboregulator #12 and pSEVA238 D.M. GFP were kind gifts from the Isaacs Lab (Yale University) and the Molecular Environmental Microbiology Laboratory (CNB-CSIC), respectively. Riboregulator #12s cassette was amplified using primers R1-R2 and cloned into a R3-R4 amplified pSEVAb23 backbone. Primers R5-R6 were then used to substitute the original promoters pLacIQ and pLtetO by the xylS/pM (R7-R8) and rhaRS/pRham (R9-R10) expression systems, which were amplified from in-house vectors, to create pSEVAb23 RR12-GFP pM-pRha. Subsequently, linearised fragments lacking both the xylS/pM and the rhaRS/pRham expression systems were created using R15-R16 for riboregulator #12 and R17-R18 for riboregulator #10, which was generated by introducing the corresponding crRNA and taRNA differences with #12 in the amplification primers. Using P7 and P10, xylS/pM and rhaRS/pRham were cloned to create pSEVAb23 RR10-GFP pM-pRha, while the use of R19-R20 allowed the construction of both pSEVAb23 RR10-GFP pRha-pM and pSEVAb23 RR10-GFP pRha-pM. To build all the versions without taRNA, R22 and R21 were used as forward primers when the crRNA promoters were either pM or pRham, respectively, combined with the reverse primer R14, that eliminated the taRNA part. To obtain the last circuit, the toehold riboregulator #2.1, the pSEVAb GFP backbone was amplified with T2 and T4, whereas the part containing the xylS/pM and rhaRS/pRham expression systems of the riboregulator #12 version 1 was obtained using T1 and T3, yielding pSEVAb23 Toehold2.1-GFP pM-pRha.

For the replacement of *gfp* with the genes encoding ScCas9, TetR and AcrIIA4, the backbones of pSEVAb23 RR12/10-GFP pM-pRha, pSEVAb23 Toehold2.1-GFP pM-pRha and pSEVA238 D.M. GFP were amplified with R24-R25, R24-T5 and D1-D2, respectively. ScCas9 was amplified from pSEVAb62-ScCas9-tracrRNA-crRNA_REP_ISPpu9 using primers S1-S2 (for the DM), S3-S4 (for both conventional riboregulators) and S5-S4 (for the toehold2.1). The TetR fragment was obtained from an in-house pSEVAb65 pTet-GFP with primers TR1-TR2 (for the DM), TR3-TR4 (for both conventional riboregulators) and TR5-TR4 (for the toehold2.1). Lastly, AcrIIA4 was amplified from an in-house pSB1C3-AcrIIA4 vector making use of A1-A2 (for the DM), A3-A4 (for both conventional riboregulators) and A5-A4 (for the toehold2.1). The constitutive pSEVAb23-AcrIIA4 was generated by amplifying the backbone pSEVAb23 with the Anderson 100 promoter attached using primers R4 and R26 and by cloning into it the AcrIIA4 sequence amplified with R27-R28 from the pSB1C3-AcrIIA4.

Finally, pSEVAb62 pTet-ScCas9 was made using primers S6-S7 which changed the native ScCas9 promoter present in pSEVAb62 ScCas9 for the pLtetO expression system, which, in this case, contained two copies of the tet operator.

### 2.3 Construction of *P. putida* GenoMine

*Pseudomonas putida* “GenoMine” is how we denominate *P. putida* KT2440 equipped with the GenoMine genetic circuit. This engineered strain was generated by introducing downstream the PP_5322 genomic locus a CRISPR cassette containing two spacers that target highly repetitive regions of the *P. putida* KT2440 genome. This site had been previously characterised as a high expression locus, in which inserts did not affect the strain’s growth rate (Chaves et al., 2020). The CRISPR cassette was introduced *via* I-SceI-mediated homologous recombination (Wirth et al., 2020). In brief, the fluorescent suicide vector pGNW crRNA_REP_ISPpu9 PP_5322 KI, which carried the CRISPR cassette between two 500-bp homology regions upstream and downstream the exact introduction location, was first transformed by electroporation into *P. putida* KT2440 cells. Cointegrates, easily identified by the green fluorescence, were subjected to a second transformation with the standard pQURE6 which contains the inducible I-SceI meganuclease. Upon 3-methylbenzoate induction, a second crossover resulted in the excision of the cointegrated pGNW followed by non-fluorescent cells which needed to be scanned for revertant (*i.e*., wild type) or mutant (*i.e*., knock in) genotype. Genomic construct was verified by Sanger sequencing after PCR amplification with NEB Q5^®^ High-Fidelity DNA polymerase. Additionally, to ensure post-integration short term stability, colonies of the GenoMine strain were always verified with colony PCR before cleavage assays.

### 2.4 Cleavage assays

Strains *P. putida* KT2440 (wild type) and *P. putida* GenoMine, either harbouring or not circuit plasmids, were used in different types of cleavage assays with ScCas9 plasmids. First, to test the efficiency of the GenoMine strategy, electro-competent *P. putida* cells were transformed with 100 ng of either the constitutive pSEVAb62-ScCas9 or the empty pSEVAb62, and subsequently plated onto LB-Gen solid agar media after 1.5 h of antibiotic-free recovery. In this first case, targeting efficiency was calculated by comparing the number of colony forming units (CFU) present in the GenoMine plates (targeting) and the number of CFU present in the corresponding wild type plates (non-targeting).

Second, to measure the cleavage survival when ScCas9 was controlled by the different genetic circuits, electro-competent wild type and GenoMine cells were transformed with 100 ng of either pSEVAb23 RR10-ScCas9, pSEVAb23 RR12-ScCas9, pSEVAb23 Toehold2.1-ScCas9 or pSEVA238 D.M. ScCas9, alongside their non-targeting counterparts carrying GFP instead of ScCas9. After 1.5 h of recovery in absence of antibiotics and inducers, 100 μL of each transformed sample was plated on LB-Kan, LB-Kan-3MB, LB-Kan-Rham and LB-Kan-3MB-Rham in the case of the riboregulators, and onto LB-Kan and LB-Kan-3MB, in the case of the digitaliser module. Cleavage survival was calculated after 48 h as the ratio between CFU growing upon different induction conditions and total CFU growing on plates without the addition of inducers and expressed in percentage.

Third, to measure cleavage survival when the circuits were controlling the expression of AcrIIA4, strains KT2440 and GenoMine were first transformed with the AcrIIA4-containing plasmid pSEVAb23-AcrIIA4. For the subsequent transformation, cultures were grown overnight under all the different inducer-combinations and then OD_600_ corrected. Then, they were transformed with 100 ng of either the constitutive pSEVAb62 ScCas9 or the empty pSEVAb62 and, in this case, recovery of each sample was performed for 1.5 h in the presence of the same inducer in which it had been previously incubated. Subsequently, 100 μL of the samples was correspondingly plated onto LB-Kan-Gen. pSEVAb62 dScCas9 was used in the same manner to ascertain the cause of the low transformation efficiencies when using a constitutive plasmid expressing ScCas9.

Fourth, to also measure cleavage survival, but this time when the circuits were controlling the expression of the TetR repressor, both strains KT2440 and GenoMine had to be transformed in advance with the TetR-containing plasmids pSEVAb23 RR10-TetR, pSEVAb23 RR12-TetR, pSEVAb23 Toehold2.1-TetR and pSEVA238 D.M. TetR. All these different strains were subsequently transformed with 100 ng of either pSEVAb62-pTet-ScCas9 or the empty pSEVAb62 and incubated for 1.5 h of recovery in the absence of antibiotics but in presence of all the inducer combinations. 100 μL of the samples was then correspondingly plated onto LB-Kan-Gen, LB-Kan-Gen-3MB, LB-Kan-Gen-Rham or LB-Kan-Gen-3MB-Rham in the case of the riboregulators, and onto LB-Kan-Gen or LB-Kan-Gen-3MB in the case of the digitaliser module.

Cleavage survival was calculated after 48 h as the ratio between CFU growing upon different induction conditions and total CFU growing on plates without the addition of inducers and expressed in percentage. Every cleavage assay was performed at least in biological triplicates.

### 2.5 Survival test from permissive conditions to non-permissive conditions

To understand the effect of transitioning from permissive to non-permissive conditions, surviving cells in permissive medium from the cleavage assays were tested again. Transfer to new plates was done directly and after a new growth phase of 24 h in liquid cultures. The experiment was performed only with cells carrying pSEVAb23 RR12-ScCas9 that had survived the cleavage assay under permissive conditions (non-induced). Three individual CFU were picked and resuspended in 100 μL of sterile water. 20 μL of the resuspension was plated on LB-Kan, LB-Kan-3MB, LB-Kan-Rham and LB-Kan-3MB-Rham. The remaining 20 μL was used to inoculate a 10 mL liquid culture of LB-Kn which was grown overnight. Next day, 20 μL of the grown culture was plates on LB-Kan, LB-Kan-3MB, LB-Kan-Rham and LB-Kan-3MB-Rham to assess the survival after a few generations. Cleavage survival was calculated after 48 h as the ratio between CFU growing upon different induction conditions and total CFU growing on plates without the addition of inducers and expressed in percentage.

### 2.6 Fluorescence assays

*Pseudomonas putida* KT2440 harboring either pSEVAb23 RR12-GFP pM-pRham, pSEVAb23 RR12-GFP pRham-pM, pSEVAb23 RR10-GFP pM-pRham, pSEVAb23 RR10-GFP pRham-pM, pSEVAb23 cr12-GFP pM-pRham, pSEVAb23 cr12-GFP pRham-pM, pSEVAb23 cr10-GFP pM-pRham, pSEVAb23 cr10-GFP pRham-pM, pSEVAb23 Toehold2.1-GFP pM-pRham or pSEVA238 D.M. GFP were grown at 30°C and 200 rpm, overnight in 10 mL LB-Kan. Overnight cells were harvested at 4,700 g for 5 min and washed twice with minimal M9 medium to eliminate LB traces. Cells were then resuspended to an OD_600_ = 0.3 in fresh M9 medium (1.63 g/L NaH_2_PO_4_, 3.88 g/L K_2_HPO_4_, 2 g/L (NH_4_)_2_SO_4_, 10 mg/L EDTA, 100 mg/L MgCl_2_.6H_2_O, 2 mg/L ZnSO_4_.7H_2_O, 1 mg/L CaCl_2_.2H_2_O, 5 mg/L FeSO_4_.7H_2_O, 0.2 mg/L Na_2_MoO_4_.2H_2_O, 0.2 mg/L CuSO_4_.5H_2_O, 0.4 mg/L CoCl_2_.6H_2_O, and 1 mg/L MnCl_2_.2H_2_O) supplemented with 50 mM of glucose and 50 mg/L kanamycin on appropriate 96-well plates for the measurement of both absorbance and fluorescence in a total volume of 200 µL per well. In addition, inducers were also provided when necessary, including L-rhamnose at 3.75 mM and 3-methylbenzoate at 2 mM. Optical density (OD_600_) and fluorescence (excitation 467 nm, emission 508 nm) were monitored over 24 h using a BioTek Synergy Mx Multi-Mode Microplate reader. Relative fluorescence values were calculated by normalising the values of fluorescence to the OD_600_ values. Two biological and three technical replicates were included.

## 3 Results

### 3.1 ScCas9 effectively targets repetitive genomic regions in *Pseudomonas putida* starting a chain reaction of chromosome cleavages that result in cell death

**FIGURE 1:**
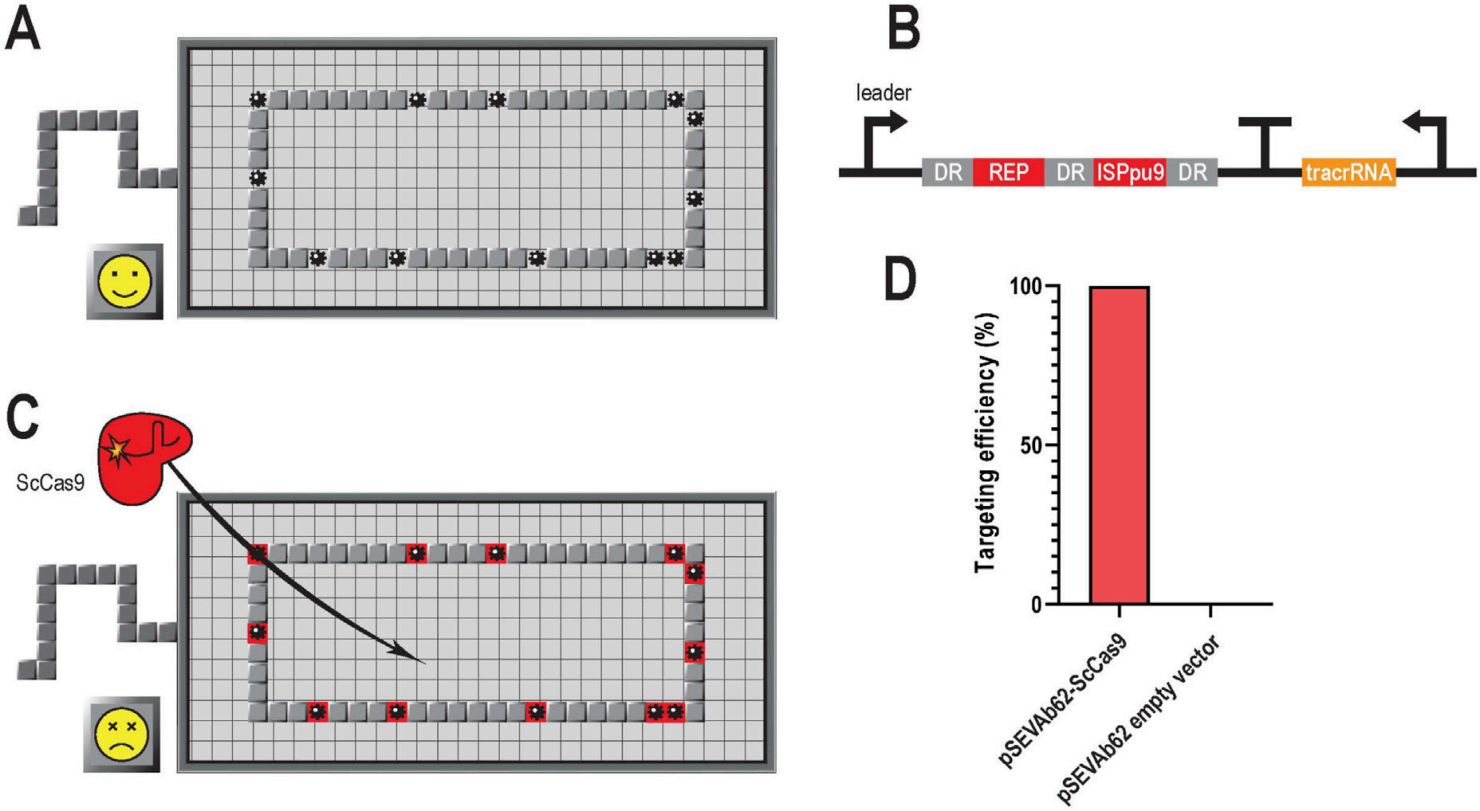
Scheme of GenoMine, a CRISPR-Cas9-based kill switch in *Pseudomonas putida* **(A)** The genome of *Pseudomonas putida* KT2440 contains abundant repetitive sequences. These repetitive regions can easily be used as CRISPR-Cas9 targets leading to a chain reaction of cleavages in the bacterial chromosome **(B)** GenoMine cassette introduced downstream the PP_5322 locus of the genome of *Pseudomonas putida* KT2440 containing a CRISPR array with two constitutively expressed spacers (REP and ISPpu9) that target repetitive regions of the genome itself. DR stands for direct repeat **(C)** When ScCas9 is transformed into cells containing the GenoMine cassette, a chain reaction of cleavages starts resulting in cell death **(D)** Experimental validation of the hypothesis depicting the targeting efficiency associated with cell death of *Pseudomonas putida* “GenoMine” relative to that obtained in the wild type strain when transformed with a pSEVAb62 plasmid constitutively expressing ScCas9 or with an empty plasmid (mean ± s.d., n = 3 biological).

The genome of *P. putida* KT2440 contains more than 900 REPs and 7 ISPpu9 sites (Gómez-García et al., 2021). Based on their highly conserved sequences, we designed two corresponding spacers of 30 nucleotides (Supplementary Table S4). Considering that ScCas9 requires a 5′-NNG-3′ PAM located downstream of the protospacer or targeted region, these two spacers could theoretically target the genome at 27 different loci that shared 100% sequence identity. However, it is estimated that the cRNA:tracrRNA:Cas9 might be able to target a much higher number of loci, due to the fact that the Cas9 complex can target sequences that differ by one or two nucleotides from the spacer, as long as these occur out of the seed sequence (the 8–10 nucleotides immediately adjacent to the PAM) (Jiang et al., 2013) (Figure 1A). Subsequently, we successfully generated the *P. putida* GenoMine strain by introducing a constitutive CRISPR cassette containing both the REP and ISPpu9 spacers between 36 bp-long direct repeats (DR) and the sequence for the tracrRNA (Figure 1B) (Supplementary Table S5), downstream the PP_5322 gene to achieve high levels of expression (Chaves et al., 2020) using I-SceI mediated homologous recombination (Wirth et al., 2020).

To test the genotoxicity of ScCas9 in the GenoMine strain (Figure 1C), we transformed it with the plasmid pSEVAb62-ScCas9, in which the endonuclease was constitutively expressed. As a result, a targeting efficiency of 99.95% ± 0.03% was achieved when compared with the wild type strain transformed with the same plasmid (Figure 1D). As a negative control, an empty pSEVAb62 plasmid was used to transform both the GenoMine and the wild type. In this case, growth of the GenoMine strain was not limited compared to the wild type strain, indicating that the former without Cas9 activity does not show growth limitations compared to the latter.

### 3.2 Utilisation of different genetic circuits to control gene expression in *P. putida*

**FIGURE 2:**
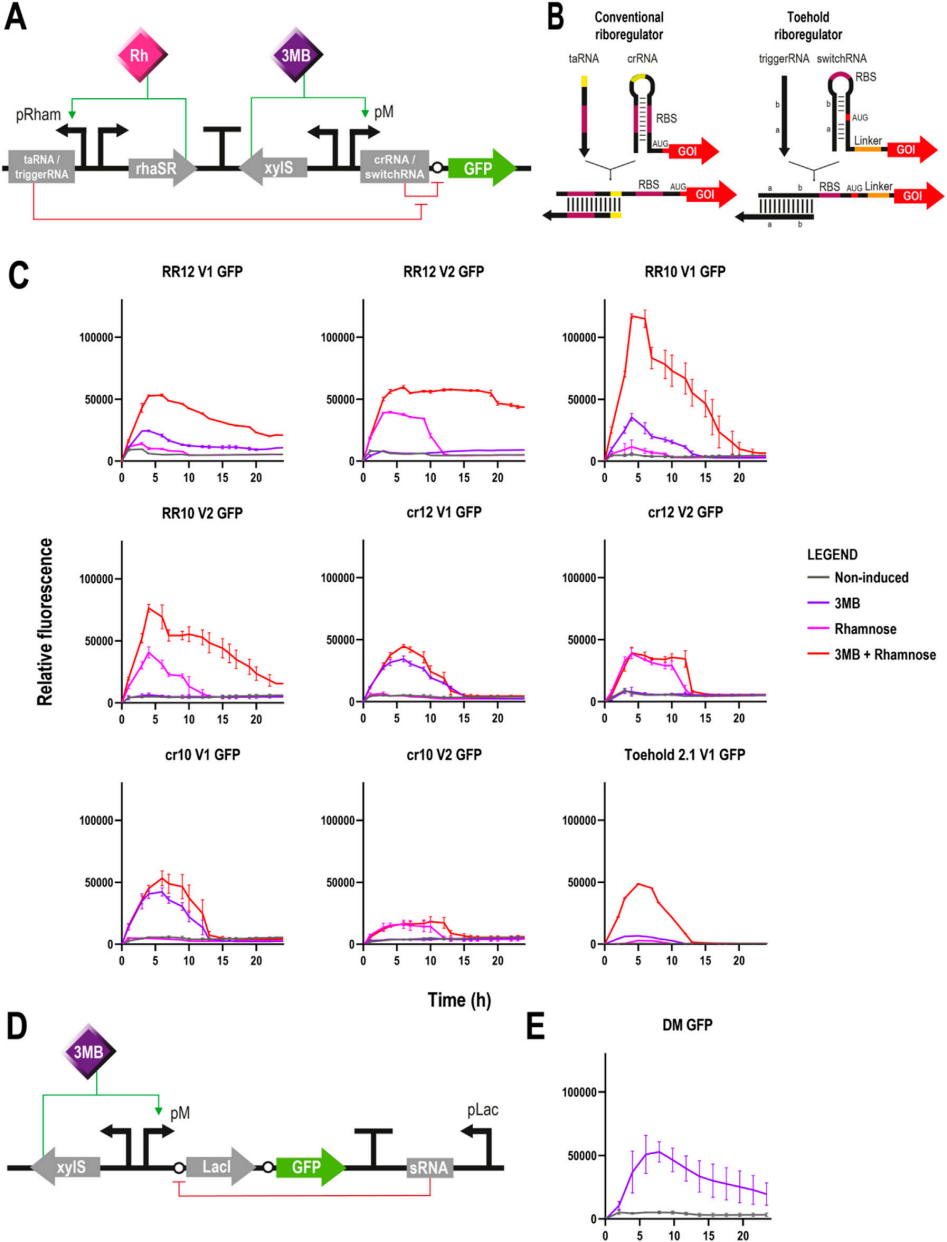
Different circuits controlling GFP expression in *Pseudomonas putida* **(A)** Schematic representation of a riboregulator circuit. Two different modules are cloned in opposite directions and separated by a transcriptional terminator: on the left, the trans-activating RNA (conventional riboregulator) or trigger RNA (toehold) molecule is controlled by the rhaRS/pRham expression system; and on the right, the cis-repressing RNA (conventional riboregulator) or switch RNA (toehold) is transcriptionally coupled to a *gfp* gene under the control of the xylS/pM expression system (Promoter version 1). The transcription of the aforementioned modules is induced by rhamnose and 3-methylbenzoate, respectively. Translation of *gfp* is blocked by the crRNA/switchRNA in absence of the taRNA/triggerRNA, but in its presence, the binding of the two RNA molecules results in a conformational change that allows the ribosome to access the RBS for *gfp* translation **(B)** Structural differences between conventional and toehold riboregulators. While the former represses translation by base pairing directly to the RBS region (pink), the latter does it through base pairs programmed before and after the AUG start codon (red), leaving this and the RBS (pink) completely unpaired **(C)** Fluorescence assays of the different riboregulator variants controlling the expression of *gfp* depicting relative fluorescence over time for 24 h. From top left to bottom right: RR12 version 1, RR12 version 2, RR10 version 1, RR10 version 2, crRNA 12 version 1, crRNA 12 version 2, crRNA 10 version 1, crRNA 10 version 2 and Toehold 2.1 version 1. Grey lines represent non-induced conditions, purple lines induction only with 3MB, pink lines only with rhamnose and red lines double induction with both 3 MB and rhamnose (mean ± s.d., n ≥ 2 biological and 3 technical replicates) **(D)** Schematic representation of the ON/OFF digitaliser module (DM). Expression of *gfp* is controlled by the xylS/pM expression system and an additional mutual inhibition circuit that regulates the translation step. This switch-like inhibition circuit includes a small RNA that inhibits the translation of the transcriptionally coupled LacI, a repressor of the pLac promoter that in turn controls the small RNA production. Activation of the system occurs only under 3MB induction, whereas in its absence, the small RNA interferes with any possible basal translation levels **(E)** Fluorescence assay of a digitalised version of the xylS/pM system controlling the expression of *gfp* depicting relative fluorescence over time for 24 h (mean ± s.d., n ≥ 2 biological and 3 technical replicates). Legend applies to panels C and E.

In order to control the triggering of the lethal cleavages, our efforts focused on finding and testing systems that would react to external signals to activate gene expression in *P. putida*. Our first approach involved the use of engineered riboregulators (RR) which operate at post-transcriptional level through small synthetic RNAs that enable gene silencing and activation. Specifically, we selected the riboregulators #12 and #10 (RR12 and RR10) from Isaacs et al. (2004) due to their effectiveness in *E. coli* (Supplementary Table S5). Their structure consists of (i) a complementary cis-sequence which binds to the ribosomal binding site (RBS) at the 5′- untranslated region (UTR) of a given gene, and consequently forms a stem-loop structure, silencing gene expression by blocking the RBS, and (ii) a small non-coding trans-activating RNA (taRNA) that acts as counterpart and targets the cis-repressing RNA (crRNA) with high affinity, enabling a conformation change which frees the RBS for the binding of the translation machinery (Figure 2A) (Isaacs et al., 2004). These two molecules were cloned into a pSEVAb23 plasmid under the control of the xylS/pM and rhaRS/pRham expression systems, induced by 3-methylbenzoate (3 MB) and L-rhamnose, respectively, to regulate the expression of a reporter *gfp* gene. In the first version of our system (V1), xylS/pM was controlling the transcription of the crRNA-*gfp* molecule whereas rhaRS/pRham was controlling the taRNA. In the second version (V2), the inducible expression systems were interchanged to evaluate the effect and potential leakiness of the promoters.

Fluorescence assays revealed the correct silencing and activation of *gfp* under the corresponding non-induced and double induction (3 MB + rhamnose) conditions using the two versions of both riboregulators (Figure 2C). Regarding RR12, very low levels of fluorescence were observed in both versions under non-induced conditions. Conversely, when both inducers were supplied, *gpf* expression levels increased dramatically, with version 2 showing a more stable and longer expression than version 1. When using RR10, fluorescence levels were insignificant under non-induced conditions. Upon double induction, RR10 showed similar profiles in both versions, albeit expression levels of version 1 were higher. When compared to RR12, both versions showed higher expression with RR10.

With respect to the individual induction with 3 MB or rhamnose, the outcomes were similar when using either RR12 or RR10. Results showed no expression (version 2) or very low GFP levels (version 1) when only the taRNA was induced. Conversely, the inducer of the crRNA molecule alone was able to yield significant levels of GFP expression (3 MB-induction for version 1 and rhamnose-induction for version 2) indicating either leaky expression of the promoters (Calero et al., 2016) or improper functioning of the crRNA molecule (Figure 2C). The first hypothesis was quickly discarded based on the results produced by the different configurations, as it would be unlikely that both the pRham and the pM promoters would exhibit the same level of leakiness. The second hypothesis regarding the functioning of the crRNA molecule was experimentally investigated with pSEVAb23 plasmids containing the crRNA-*gfp* module but lacking the taRNA part.

Expression of *gfp* was observed with the crRNA part of both RR12 and RR10 and with their corresponding two versions despite the absence of taRNA within the system. In this case, relative fluorescence levels with the crRNA inducer only and with both inducers were very similar and consistent with those of the previous assays when only the inducer of the crRNA was provided (Figure 2C). These results showed that *gfp* translation was still possible even without taRNA, indicating that the cis-repressor sequence did not manage to block completely the access of the ribosome to the *gfp*’s RBS.

In an attempt to solve this issue and move away from relying on the RBS sequence, we tested the toehold riboregulator approach. The principle of a toehold switch is based on the design of two RNA sequences flanking the start codon that match and form a hairpin with another complementary RNA sequence located before the RBS, which is left completely unpaired in this configuration (Figure 2B). Because neither the RBS nor the start codon are necessary for hairpin formation, the crRNA can have any arbitrary sequence instead of a limited amount of them, which allows greater riboregulator variability. In this case, the reconfigured crRNA sequence is called switch RNA, whereas the equivalent of the former taRNA receives the name of trigger RNA (Green et al., 2014). For our study, we selected the toehold switch #1 of the second- or forward engineering-generation (#2.1) of Green et al. (2014) which had previously shown the highest ON/OFF GFP fluorescence ratio (665 ± 135) in their study using *E. coli* (Supplementary Table S5). Toehold riboregulator #2.1 (Toehold 2.1) was cloned into a pSEVAb23 vector controlling a *gfp* reporter gene and, this time, only version 1, meaning switchRNA-*gfp* under the control of xylS/pM and triggerRNA controlled by rhaRS/pRham, was generated. In this case, the fluorescence assay using the Toehold 2.1 reported a correct functioning of the circuit under non-induced and double induction conditions. Unfortunately, undesired *gfp* expression levels were observed when only the switchRNA inducer was provided, as it had previously happened with RR12 and RR10 (Figure 2C). However, these one-inducer activation levels were considerably lower than those obtained with the conventional riboregulators in the same conditions (Figure 2C).

With the objective of minimising the levels of gene expression under non-induced conditions, our third approach was the use of an ON/OFF digitaliser module (DM) that supresses the basal level of promoters entirely, impeding gene expression in the absence of induction. The rationale of this circuit is based on the interplay of a translation-inhibitory small RNA controlled by pLac with the translational coupling of the gene of interest to the repressor LacI (Figure 2D) (Calles et al., 2019). Instead of an AND gate like the riboregulators that theoretically require the presence of both inducers for the expression of a gene, the ON/OFF DM needs only a single input to accomplish a tight control of the system (YES gate). Even though it had already been tested in *P. putida* using the xylS/pM expression system and GFP as reporter (Calles et al., 2019), we repeated the experiment and obtained results consistent with previous research (Figure 2E). Basal fluorescence levels were minimal while expression under 3 MB-induced conditions were similar to those achieved with double induced RR12, version 1 (Figure 2C).

### 3.3 Induced conditions activate ScCas9 resulting in cell death

**FIGURE 3:**
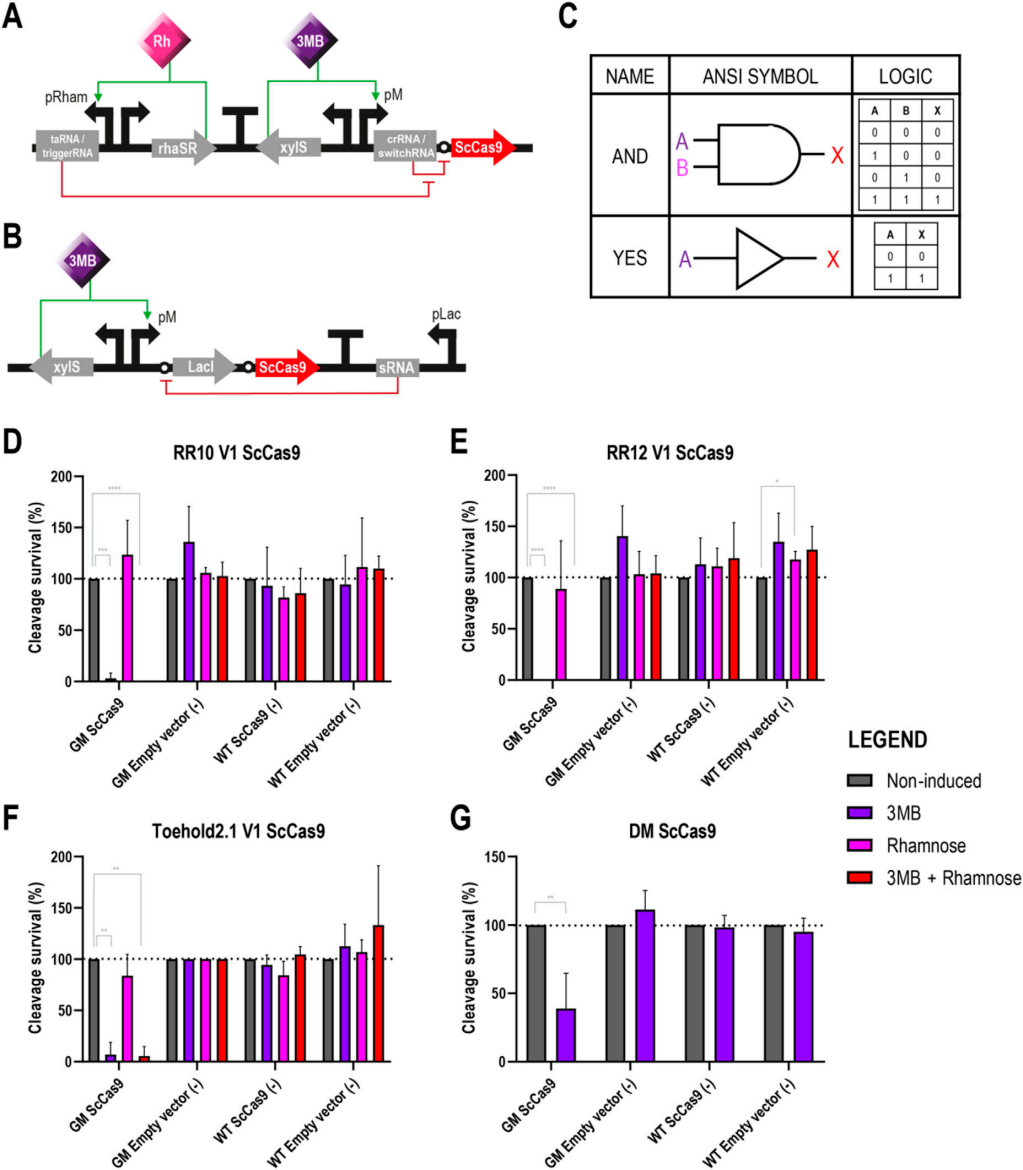
Different circuits controlling the expression of ScCas9 in *Pseudomonas putida* GenoMine **(A)** Schematic representation of the riboregulator circuits (conventional or toehold) controlling the expression of ScCas9 **(B)** Schematic representation of the xylS/pM expression system endowed with the ON/OFF digitaliser module and controlling the expression of ScCas9 **(C)** Designs of AND YES gates according to the American National Standards Institute (ANSI), in this case corresponding to the riboregulators and the ON/OFF DM, respectively. Following binary logic, there are only two states allowed, 1 and 0 or “on and off”. A and B represent the inputs 3MB and rhamnose, and X represents the output of the circuit, in this case the activation of Cas9 **(D)** Cleavage survival after transformation of ScCas9 under the control of conventional RR10 **(E)** Cleavage survival after transformation of ScCas9 under the control of conventional RR12 **(F)** Cleavage survival after transformation of ScCas9 under the control of Toehold 2.1 **(G)** Cleavage survival after transformation of ScCas9 under the control of a digitalised version of the xylS/pM system. Relative cleavage survival was calculated separately for each strain in each figure as the ratio between CFU growing upon different induction conditions (purple bars represent single induction with 3MB, pink bars single induction with rhamnose and red bars double 3MB + rhamnose induction) and total CFU growing on plates without the addition of inducers (grey bars) and expressed in percentage. Only significant values are indicated for a parametric two-tailed t-test between two groups, where *p < 0.05; **p < 0.01; ***p < 0.001; and ****p < 0.0001; non-significant values were not depicted (mean ± s.d., n ≥ 3 biological).

To determine whether controlled induction of the different systems could result in cell death in the GenoMine strain, we replaced in all four circuits the reporter *gfp* with the *sccas9* gene which had previously been codon optimised for *P. putida* (Asin-Garcia et al., 2021). For convenience and given the scarce behavioural differences between the two configurations, only version 1 of the riboregulators was used in subsequent experiments (Figure 3A). Plasmids pSEVAb23 containing these constructs (Figures 3A, B), alongside an empty pSEVAb23 vector were transformed in both wild type and GenoMine *P. putida* strains, which were subsequently plated on media supplemented with the different inducers to quantify the cell population survival to the CRISPR-Cas9 lethal response. We used for each strain- and plasmid-transformation the number of colony forming units present in the non-induced plates as the reference of 100% survival.

Upon full induction of the genetic circuits, which is 3 MB + rhamnose for the riboregulators (AND gate) and 3 MB only for the ON/OFF DM (YES gate) (Figure 3C), the endonuclease should be expressed leading to the chromosome’s cleavage of the strain carrying the GenoMine CRISPR cassette. Conversely, in the three control cases lacking any of the CRISPR-Cas9 machinery elements, namely, the two transformations of the wild type and the transformation of the empty vector into the GenoMine strain, cleavages were not expected. Accordingly, the obtained number of CFU in the three negative controls was generally not affected by the different inducer conditions, apart from a single exception (WT strain transformed with an empty RR12 vector upon rhamnose induction, Figure 3E) (Figures 3D–G).

On the other side, we had our test situation when the GenoMine strain was transformed with vectors carrying ScCas9. Upon fully induced conditions, there was a strong decrease in the percentage of surviving cells with all four circuits. When the endonuclease was under the control of the conventional riboregulators, the levels of lethality under fully induced conditions were absolute, with no CFU present in the plates. When using the toehold, 5.39% ± 9.33% survived, whereas 38.97% ± 25.66% of the CFU managed to endure the cleavage with the DM (Figures 3D–G). In absence of 3MB, upon single induction of the taRNA/triggerRNA riboregulator counterparts with rhamnose, the cleavage survival was restored to levels that did not significantly differ from those of the non-induced conditions (Figures 3D–F). However, as we had previously seen with the GFP, activation of ScCas9 still occurred upon single 3 MB induction of the crRNA/switchRNA molecules. Percentages of cleavage survival dropped to 3.03% ± 5.24%, 0% and 6.86% ± 11.88% when ScCas9 was controlled by RR10, RR12 or toehold 2.1, respectively (Figures 3D–F).

While Figures 3D–G depict relative data calculated separately for each strain and transformation, we must remark that the absolute numbers in the obtained plates were variable (Supplementary Table S6). It should be noted that the absolute CFU count of non-induced GenoMine and wild type strains transformed with ScCas9 under the control of the different circuits was heavily impacted compared to the CFU count of GenoMine strains transformed with an empty vector (Supplementary Table S6). This indicated that there was either: (i) ScCas9-activity under non-induced conditions, which could be attributed to leakiness of the circuits that are in control of ScCas9 expression, or (ii) a significant burden or toxicity derived from hosting plasmids containing the endonuclease, even when this does not result in any chromosomal cleavage. To quantify this effect, we calculated the CFU difference between the wild type strain transformed with either the ScCas9-expressing plasmid or the same plasmid containing a catalytically inactive version of ScCas9 (dScCas9) with the empty plasmids (Supplementary Figure S2). As we had already seen in Figure 1D, the lethal effect of ScCas9 was almost absolute in the GenoMine strain. However, in the wild type strain, the presence of ScCas9, either catalytically active or not, also places a substantial burden that results in reduced cell populations, even without mediating any cleavage. The fact that this result is also observed with the catalytically inactive version indicates that this is an effect of protein burden, rather than off target effects.

To complete this part, a short-term survival test was conducted to understand the effect of transitioning from permissive to non-permissive conditions. Cells carrying ScCas9 under the control of conventional riboregulator RR12 were selected for this assay. Cells that had survived under permissive (non-induced) conditions were tested again in new test plates immediately and after 24 h of growth in liquid LB-Kn medium. Directly re-assessed cells exhibited behaviour very similar to that observed after the first exposure to the test conditions, as depicted in Figure 3E (Supplementary Figure S1). This result indicates that the GenoMine strategy remains active when transferred from permissive to non-permissive conditions. However, after a few generations and 24 h of growth in liquid permissive conditions, the functionality of the circuit is affected, and the genotoxic effect of the kill switch seems to decrease, resulting in a higher number of survivors growing under non-permissive conditions (Supplementary Figure S1).

### 3.4 Controlling *P. putida* GenoMine through ScCas9 DNA-binding inhibition and transcriptional repression

In order to develop a kill switch that is activated under non-induced conditions (*i.e.,* non-permissive conditions) instead of induced conditions, we attempted two different strategies: ScCas9’s DNA-binding inhibition using the anti-CRISPR (Acr) protein AcrIIA4, and ScCas9’s transcriptional repression using the repressing TetR/pLtetO expression system. These new elements would work as inverter devices to reverse the circuits’ outcome and logic, from AND and YES gates, in which ScCas9 expression was only possible in case of a full induction (Figure 3C), to NAND and NOT gates, in which ScCas9 expression should happen under any other scenario that is not full induction (Figure 5D).

**FIGURE 4:**
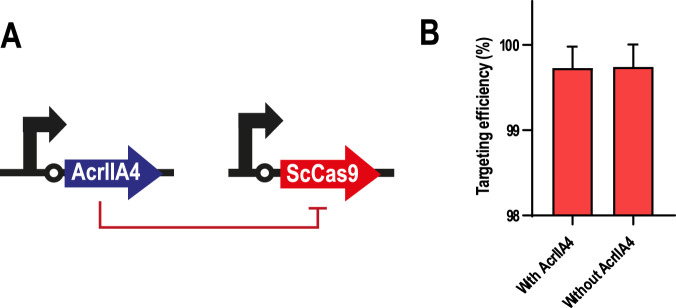
AcrIIA4 as a repressor of ScCas9 in *Pseudomonas putida* GenoMine **(A)** Schematic representation of the inhibition design **(B)** AcrIIA4 does not inhibit ScCas9’s cleavage ability in the GenoMine strain. Targeting efficiency was calculated in *Pseudomonas putida* GenoMine using the ratio of CFU obtained when transformed with either pSEVAb62-ScCas9 (targeting conditions) or an empty pSEVAb62 (non-targeting conditions), both in the presence and in the absence of a constitutively expressed AcrIIA4 (mean ± s.d., n = 2 biological).

In our first strategy, we tested if AcrIIA4 could effectively inhibit ScCas9, as had been previously reported for SpyCas9 (Dong et al., 2017; Yang and Patel, 2017). For this, we first transformed the GenoMine strain with pSEVAb23-AcrIIA4, in which the anti-CRISPR protein was constitutively expressed. Secondly, pSEVAb62-ScCas9 was also introduced to evaluate AcrIIA4’s efficacy to prevent the action of ScCas9. The outcome of this experiment showed however no effect of AcrIIA4. Either in the presence or in the absence of this inhibitor, ScCas9 was equally able to target 99.73% ± 0.25% and 99.74% ± 0.25% of the respective cell populations, when compared with those transformed with an empty pSEVA62 vector (Figure 4). As a consequence, we decided not to continue with this approach and attempted an alternative way to repress ScCas9’s activity.

**FIGURE 5:**
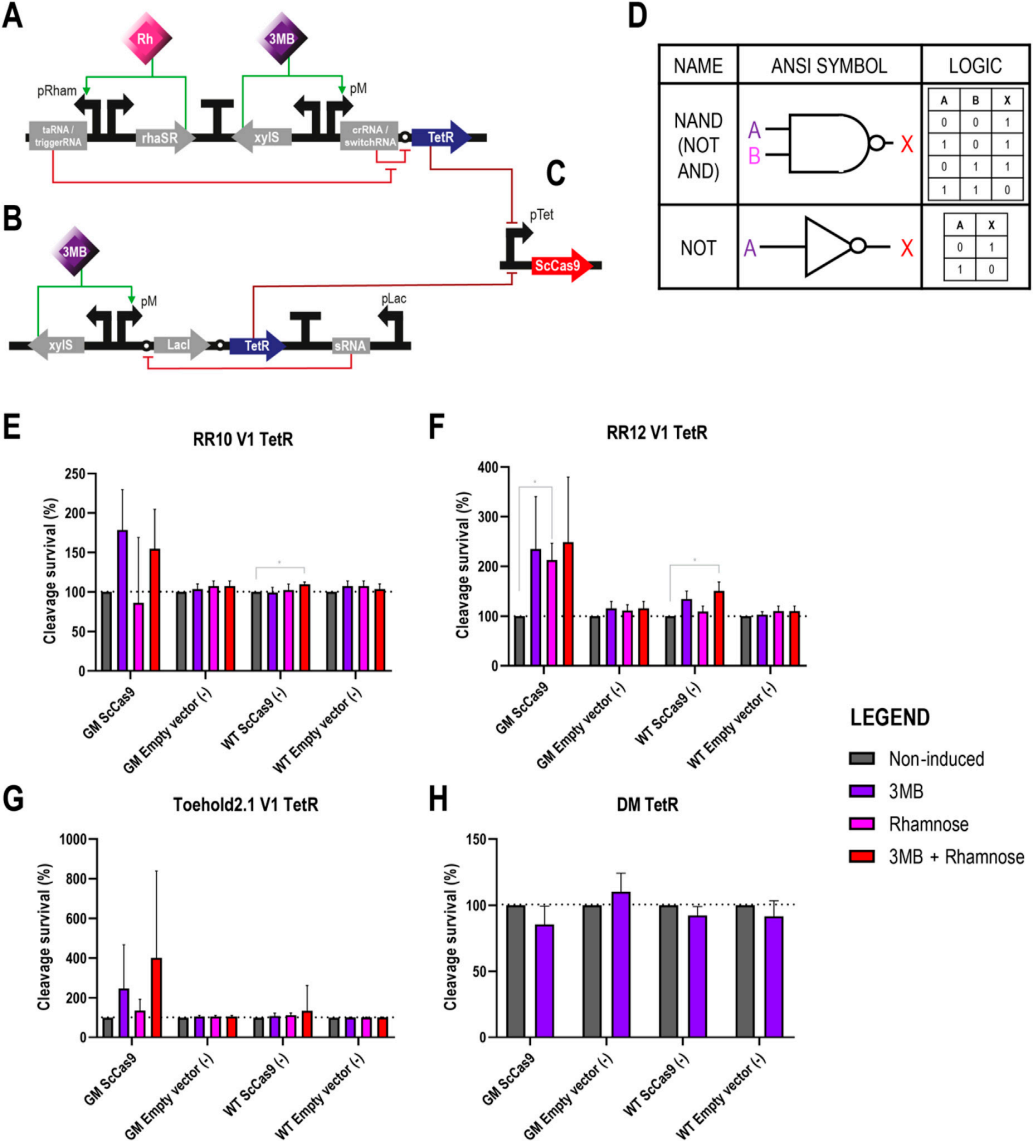
Different circuits controlling the expression of TetR, which works as a transcriptional repressor of ScCas9 in *Pseudomonas putida* GenoMine **(A)** Schematic representation of the riboregulator circuits (conventional or toehold) controlling the expression of TetR **(B)** Schematic representation of the xylS/pM expression system endowed with the ON/OFF digitaliser module and controlling the expression of TetR **(C)** ScCas9 located on a separated plasmid under the control of the TetR/pLtetO expression system. ScCas9 can be repressed in the presence of TetR, which, in turn, gets expressed upon full induction of the genetic circuits depicted in either **(A)** or **(B) (D)** Designs of NAND (NOT + AND) and NOT gates according to the American National Standards Institute (ANSI), in this case corresponding to the riboregulators and the ON/OFF DM, respectively. Following binary logic, there are only two states allowed, 1 and 0 or ‘on and off’. A and B represent the inputs 3MB and rhamnose, and X represents the output of the circuit, in this case the activation of TetR and, correspondingly, the repression of ScCas9 **(E)** Cleavage survival of cells bearing TetR under the control of RR10 after transformation with pLtetO-ScCas9 **(F)** Cleavage survival of cells bearing TetR under the control of conventional RR12 after transformation with pLtetO-ScCas9 **(G)** Cleavage survival of cells bearing TetR under the control of Toehold 2.1 after transformation with pLtetO-ScCas9 **(H)** Cleavage survival of cells bearing TetR under the control of a digitalized version of the xylS/pM expression system after transformation with pLtetO-ScCas9. Relative cleavage survival was calculated separately for each strain in each figure as the ratio between CFUs growing upon different induction conditions (purple bars represent single induction with 3MB, pink bars single induction with rhamnose and red bars double 3MB + rhamnose induction) and total CFUs growing on plates without the addition of inducers (grey bars) and expressed in percentage. Only significant values are indicated for a parametric two-tailed t-test between two groups, where *p < 0.05; **p < 0.01; ***p < 0.001; and ****p < 0.0001; non-significant values were not depicted (mean ± s.d., n = 3 biological).

In our second strategy, we substituted the *sccas9* gene for the one encoding the transcriptional repressor TetR in all four circuits (Figures 5A, B). In turn, *sccas9* was expressed on a separate plasmid under the control of the pLtetO promoter, whose activity can be repressed by binding of TetR (Saenger et al., 2000) (Figure 5C). Plasmids pSEVAb23 carrying the TetR circuit constructs were first transformed into the wild type and GenoMine strains as we had done with the ScCas9 circuit constructs in the previous assay. This time, however, a second transformation using either pSEVAb62-pLtetO-ScCas9 or an empty pSEVAb62 vector was performed, followed by an evaluation of the cleavage survival under the different inducer combinations. The number of CFU accounted under non-induced conditions represented a 100% survival for each strain- and plasmid-transformation. It is important to consider that this survival, however, is the result of an activated ScCas9 with the power of killing the GenoMine cells. Upon induced conditions, TetR should be activated repressing ScCas9’s transcription, resulting in a larger number of CFU and therefore cleavage survival levels higher than 100%.

Once again, in the negative controls with an incomplete CRISPR-Cas9 system (wild type strains and GenoMine transformed with the empty vector), the addition of the inducers did not significantly affect the cleavage survival except in a few cases (Figures 5E–H). This time, however, inducers had no significant effect on the cleavage survival of the test strain either (GenoMine transformed with ScCas9).

In this way, the GenoMine strain carrying ScCas9 did not show a significantly higher cleavage survival when the transcriptional repressor TetR was expressed under fully induced conditions of any of the circuits. Even though the highest increases in average cleavage survival were observed upon induction of the riboregulators in this strain, the large standard deviations made the results not significantly different from the non-induced conditions (Figures 5E–G). Single induction of the crRNA/switchRNA molecules resulted again in activation of the riboregulator systems, also boosting the average cleavage survival although in a non-significant manner (Figures 5E–G). In addition, single induction of the taRNA when using RR12 reported a significant cleavage survival that reached 212.9% ± 34.04% (Figure 5E). Lastly, when assessing TetR under the control of the digitalised version of the xylS/pM expression system, induced conditions did not contribute to increases in cleavage survival at all (Figure 5H).

Relative data illustrated in Figure 5 was calculated separately for each strain and transformation. As aforementioned for the cleavage assays depicted in Figure 3, the absolute CFU numbers in the obtained plates were variable (Supplementary Table S6), with a dramatic reduction of CFU in strains transformed with the ScCas9-expressing plasmid.

## 4 Discussion

This study describes the generation of a CRISPR-Cas9-mediated biocontainment strategy in *P. putida* and details direct and indirect mechanisms of transcriptional, post-transcriptional and post-translational control over the system. After knocking in a genotoxic CRISPR array that targets repetitive genomic elements, cell viability becomes challenged in the presence of a Cas9 protein. In this strain, equipped with the kill switch that we named GenoMine, the powerful lethal effect arises from the large collection of loci spread across the chromosome susceptible to the targeted cleavage (Figure 1). To trigger this chain reaction, Cas9 was configurated by means of kill switches that included some genetic regulatory elements never used before in *P. putida*.

When using engineered riboregulators, we found that conformational changes in RNA structures and corresponding intermolecular crRNA-taRNA and switchRNA-triggerRNA interactions resulted in the expected RBS exposition that unleashes translation (Figures 2B, C) (Isaacs et al., 2004; Green et al., 2014). However, flawed or incomplete repressive ability of the 5′-UTR cis elements of the crRNA and switchRNA molecules allowed undesired access of the ribosome to the RBS and subsequent post-transcriptional gene expression (Figure 2C). The cis RNA molecules of the different riboregulators used in this study (RR10, RR12 and Toehold 2.1) had previously shown silencing levels >98% derived from translational repression (Isaacs et al., 2004; Green et al., 2014). These experiments were performed *in vivo* at intermediate transcription rate in *E. coli*. While some silencing effect was exhibited, the leaky levels of gene expression that could be observed with the fluorescence data (Figure 2C) were high enough to obtain significantly similar outcomes when inducing only the cis RNA molecule, or both the cis and trans modules of the kill switches in the GenoMine strain (Figures 3, 5).

This behavioural difference of the cis elements of both conventional and toehold riboregulators between *E. coli* and *P. putida* and the common outcome reached by all three choices applied in this study (GFP, ScCas9 and TetR) suggest two things: (i) some degree of independence from the specific nucleotide sequences, and conversely, (ii) an influence of host-specific factors on crRNA and switchRNA loop conformation and stability as the cause of this phenomenon. Because the intermolecular RNA interactions rely on specific RNA structures, the utilised cis RNA sequences were initially generated based on appropriate secondary structure predictions (Isaacs et al., 2004; Green et al., 2014). While temperature is a function of the equilibrium constant ΔG_crR-crR_ and the optimal growth temperature is different for the two organisms in question, our Mfold analysis (Zuker, 2003) revealed an even lower value of ΔG_crR-crR_ at 30°C than at 37°C, indicating that these structures should be even more stable at *P. putida*’s growth conditions than at *E. coli*’s. On another note, host specific small RNAs (Prévost et al., 2007; Sonnleitner et al., 2011) or even RNA binding proteins that typically cause translational inhibition such as Crc (Moreno et al., 2007; Moreno et al., 2009) and Hfq (Sonnleitner and Bläsi, 2014) might interfere with the hybridisation loop of the crRNA and switchRNA molecules. Specifically, these proteins could have bound to the A-rich sequence upstream the cis elements of the riboregulators in the used vectors, since it has been shown that they bind to this type of motifs in *Pseudomonas* (Moreno et al., 2015; Hernández-Arranz et al., 2016). Even though the issues seem to originate from other intrinsic factors, further sequence- and structure-based efforts to characterise novel small RNAs (Zadeh et al., 2011; Apura et al., 2020; Pobre et al., 2020) in *P. putida* could help identifying cis repressing sequences that provide a robust and complete suppression of post-transcriptional expression.

Without the proper functioning of the cis repressing elements, the logic of the AND and NAND gates of the engineered riboregulators evolved into an ON/OFF mechanism (YES and NOT gates, respectively), similar to that of conventional inducible promoters. From the GenoMine strategy standpoint, this would not need to negatively affect their mission, as long as they would be able to offer a close to ON/OFF response when transitioning from non-induced to induced conditions. Key considerations in this regard would be: (i) high induced expression levels, and (ii) very low basal expression when no inducer is provided. These two properties were also accredited to the digitaliser module, which, unlike the riboregulators, had previously been evaluated in *P. putida* (Calles et al., 2019).

When controlling ScCas9, riboregulators showed high induced expression levels resulting in the desired complete (RR10 and RR12) or almost complete (Toehold 2.1) elimination of the cell population (Figures 3D–F). Conversely, while the GFP expression produced under the DM’s control (Figure 2E) seemed in accordance with prior results, the ScCas9 levels yielded under induced conditions of the digitalised xylS/pM were only able to kill off 61% of the GenoMine cell population (Figure 3G). Things get more complicated, however, when considering the basal expression when no inducer is provided. Even though the OFF state of the DM had previously shown zero transcription and the GFP assays depicted in Figure 2C reported very low expression levels when the riboregulators were not induced, ScCas9 had a strong effect on the population in absence of inducers. Since this influence was also observed in the strain lacking the CRISPR module, and when using a catalytically inactive version of ScCas9 (Supplementary Figure S2), we hypothesised that it occurs as a result of plasmid burden and ScCas9’s own toxicity (Jakočiūnas et al., 2016; Wu et al., 2016). Additionally, this may also hint that any level of leakiness no matter how small, can be very detrimental to the cell population. Another limitation of this system is the intrinsic instability of genetic circuits, particularly, the loss of functionality of the CRISPR-Cas9 mechanism due to homologous recombination between the direct repeats or mutations in one of the CRISPR-Cas9 elements. The short-term stability of the circuit was confirmed through sequencing prior to experimentation, colony PCR before cleavage assays, and subsequent exposure of surviving cells under permissive conditions to non-permissive conditions. The latter demonstrated that our kill switch maintained its functionality after one generation but began to lose it after an increasing number of generations (Supplementary Figure S1). This phenomenon is well known in the CRISPR community (Jiang et al., 2013; Aparicio et al., 2017; Azpiroz and Laviña, 2017; Asin-Garcia et al., 2021), but this long-term instability remains the biggest limitation of the GenoMine kill switch and a crucial focus for follow-up research on the evolution of genetic circuits.

Moving on to the control over TetR, the basal expression in absence of inducers stopped being an element of concern since leaky expression of the repressor would not significantly harm the functioning of the kill switches. Instead, the high induced expression levels became even more important given the crucial task that the repressors had of blocking the action of every ScCas9 protein. Unfortunately, the induced levels of TetR by means of any of the circuits were not sufficient to inhibit the pLtetO-mediated transcription of ScCas9 (Figures 5E–H).

After the unsuccessful attempt to control ScCas9’s response by inhibiting its DNA-binding with AcrIIA4 and by repressing its transcription with TetR, we are in need of more powerful repressing systems. This concerns a set of elements that is quite scarce in *P. putida* (Martin-Pascual et al., 2021), but that is strongly needed for the well-functioning of the GenoMine strategy. When we attempted the use of anti-CRISPR, we hypothesised that AcrIIA4 would effectively inhibit ScCas9, based on the effective inhibition of SpyCas9 by AcrIIA4 (Dong et al., 2017; Yang and Patel, 2017) and the fact that ScCas9 and SpyCas9 share a sequence similarity of 89.2%. However, the fact that the PAM-specificity of ScCas9 is different from SpCas9, and that AcrIIA4 acts preventing PAM-recognition, might be the factor that negatively influenced interactions between ScCas9 and AcrIIA4 (Figure 4). Regarding the transcriptional repression, we believe that the levels of TetR that were generated upon induction were simply not enough to completely inhibit ScCas9’s transcription, which, as we have explained, is able to significantly reduce cell population at very low expression levels. Consequently, alternative repressors with capabilities to work as genetic inverters by totally inhibiting ScCas9 need to be investigated to be placed under the control of genetic devices.

While heterologous circuits are typically designed and assumed to be orthogonal (no direct genetic crosstalk interaction) to the host cell genetic background (Wang et al., 2011; Moon et al., 2012), the performance of the different genetic networks used in this study shows otherwise. Most of the presumed orthogonal components and circuits have not been experimentally tested for their effects on different hosts’ genetic machinery (Liu et al., 2018). The original configuration of the riboregulators included the lacI/pLacIQ and tetR/pLtetO inducible expression systems for the transcriptional control of the RNA molecules (Gallagher et al., 2015). However, these two systems had shown erratic behaviour in previous *P. putida* studies (Martin-Pascual et al., 2021): an excess of leakiness in the case of the lacI/pLacIQ system, and an inconsistent ability of anhydrotetracycline (aTc) to inhibit TetR repression over pLtetO. The latter fact prevented us from properly using the whole (aTc)/tetR/pLtetO system as an inducible promoter, but still allowed us to use TetR as a repressor. Overall, these reasons motivated us to substitute them by the well-established xylS/pM and rhaRS/pRham. In addition, the xylS/pM system endowed with the ON/OFF digitaliser device had performed effectively when controlling the toxic antibacterial colicin E3 in *P. putida* (Calles et al., 2019). These circumstances evince, as many other investigations before (Wang et al., 2011; Cardinale et al., 2013; Bradley and Wang, 2015; Borkowski et al., 2016), that new configurations and bacterial hosts in the former case, and new controlled genes in the latter, can affect the final outcome of heterologous gene networks. Additionally, previous research has shown that circuit plasmid copy number plays an important role in host gene expression and dramatically affects orthogonality, burden and functionality of heterologous circuits (Liu et al., 2018). This key factor did not receive special attention during the course of this study since we consistently used medium copy number plasmids for the expression of the circuits. Nonetheless, it should be noted that for a genetic system where tight regulation is required to improve stability, even consistently using medium copy number plasmids may introduce too much stochastic variation between individual cells and across generations. In the future, more attention should be paid to achieving a balanced system behaviour by considering multiple compatible plasmids with different copy numbers to address the requirements of the GenoMine circuit in *P. putida* (Wang et al., 2011; Nielsen et al., 2016). Design of Experiments (DoE) would be a valuable tool for such a study. Nonetheless, the ideal scenario would be the one in which the whole system is integrated in the genome.

Apart from the ones contemplated in this study, other regulatory elements and genetic circuits could be considered to modulate the genotoxic response of the GenoMine strategy. Especially attractive would be the aforementioned exploration of gene expression repressing elements, and new mechanisms for inducible control over CRISPR-Cas system (Cañadas et al., 2019; Patinios et al., 2021; Zhao et al., 2021; Mecacci et al., 2023), given that the key element for the well-functioning of GenoMine is precisely the tight control of the cleavage performed by Cas9.

Once its elements are properly optimised, the GenoMine kill switch would pose an excellent strategy for biocontainment studies in *P. putida* since it brings together an effective killing mechanism and a modular sensing part that allows the characterisation of new genetic regulatory elements and circuits in this bacterium. While the general challenges of efficiency, stability and robustness typically associated to genetic circuits (Xia et al., 2019; Arnolds et al., 2021) keep being present, we aimed at accelerating the circuit’s response by using two different spacers with the ability of targeting multiple genomic loci. This is expected to reduce the time window for mutations and reorganisations, including recombination between CRISPR array direct repeats or deletion of CRISPR-Cas9 elements, that might end up inactivating the pressure, being this one of the vulnerable points of the circuit as mentioned above. In addition, industrial strains equipped with the GenoMine kill switch could be considered safe in a broader sense since, once optimised, they would have the ability to prevent any further genomic alteration made with CRISPR-Cas9-based editing technologies. Thus, this host might eventually be used as an intellectual property strain to prevent industrial espionage (Cai et al., 2015; Caliando and Voigt, 2015; Kim and Lee, 2020), to biologically store sensible information, and to use in any situation in which further modifications are undesired since it offers protection against any editing that involves Cas9. These industrially attractive features built upon the value of biosafety would contribute to the realisation of a more thorough, and ultimately more legitimised, safe-by-design approach in which the properties of the biocontainment strategy itself can also be profitable in other aspects of the industrial process or final application context (Asin-Garcia et al., 2023).

## Data Availability

The original contributions presented in the study are included in the article/Supplementary Material, further inquiries can be directed to the corresponding author/s.

## References

[B1] Amarelle V. Sanches-Medeiros A. Silva-Rocha R. Guazzaroni M.-E. (2019). Expanding the toolbox of broad host-range transcriptional terminators for Proteobacteria through metagenomics. ACS Synth. Biol. 8, 647–654. 10.1021/acssynbio.8b00507 30943009

[B2] Anders C. Niewoehner O. Duerst A. Jinek M. (2014). Structural basis of PAM-dependent target DNA recognition by the Cas9 endonuclease. Nature 513, 569–573. 10.1038/nature13579 25079318 PMC4176945

[B3] Aparicio T. de Lorenzo V. Martínez-García E. (2017). CRISPR/Cas9-based counterselection boosts recombineering efficiency in *Pseudomonas putida* . Biotechnol. J. 1700161, e1700161. 10.1002/biot.201700161 29058367

[B4] Aparicio T. Nyerges A. Martínez-García E. de Lorenzo V. (2020). High-efficiency multi-site genomic editing of *Pseudomonas putida* through thermoinducible ssDNA recombineering. iScience 23, 100946. 10.1016/j.isci.2020.100946 32179472 PMC7068128

[B5] Apura P. Saramago M. Peregrina A. Viegas S. C. Carvalho S. M. Saraiva L. M. (2020). Tailor-made sRNAs: a plasmid tool to control the expression of target mRNAs in *Pseudomonas putida* . Plasmid 109, 102503. 10.1016/j.plasmid.2020.102503 32209400

[B6] Aranda-Olmedo I. Tobes R. Manzanera M. Ramos J. L. Marqués S. (2002). Species-specific repetitive extragenic palindromic (REP) sequences in *Pseudomonas putida* . Nucleic Acids Res. 30, 1826–1833. 10.1093/nar/30.8.1826 11937637 PMC113213

[B7] Arnolds K. L. Dahlin L. R. Ding L. Wu C. Yu J. Xiong W. (2021). Biotechnology for secure biocontainment designs in an emerging bioeconomy. Curr. Opin. Biotechnol. 71, 25–31. 10.1016/j.copbio.2021.05.004 34091124

[B8] Asin-Garcia E. Martin-Pascual M. Garcia-Morales L. van Kranenburg R. Martins dos Santos V. A. P. (2021). ReScribe: an unrestrained tool combining multiplex recombineering and minimal-PAM ScCas9 for genome recoding *Pseudomonas putida* . ACS Synth. Biol. 10, 2672–2688. 10.1021/acssynbio.1c00297 34547891 PMC8524654

[B9] Asin-Garcia E. Robaey Z. Kampers L. F. C. Martins Dos Santos V. A. P. (2023). Exploring the impact of tensions in stakeholder norms on designing for value change: the case of biosafety in industrial biotechnology. Sci. Eng. Ethics 29, 9. 10.1007/s11948-023-00432-6 36882674 PMC9992083

[B10] Azpiroz M. F. Laviña M. (2017). Analysis of RecA-independent recombination events between short direct repeats related to a genomic island and to a plasmid in *Escherichia coli* K12. PeerJ 5, e3293. 10.7717/peerj.3293 28503377 PMC5426353

[B11] Batianis C. Kozaeva E. Damalas S. G. Martín‐Pascual M. Volke D. C. Nikel P. I. (2020). An expanded CRISPRi toolbox for tunable control of gene expression in *Pseudomonas putida* . Microb. Biotechnol. 13, 368–385. 10.1111/1751-7915.13533 32045111 PMC7017828

[B12] Bikard D. Barrangou R. (2017). Using CRISPR-Cas systems as antimicrobials. Curr. Opin. Microbiol. 37, 155–160. 10.1016/j.mib.2017.08.005 28888103

[B13] Borkowski O. Ceroni F. Stan G.-B. Ellis T. (2016). Overloaded and stressed: whole-cell considerations for bacterial synthetic biology. Curr. Opin. Microbiol. 33, 123–130. 10.1016/j.mib.2016.07.009 27494248

[B14] Bradley R. W. Wang B. (2015). Designer cell signal processing circuits for biotechnology. New Biotechnol. 32, 635–643. 10.1016/j.nbt.2014.12.009 PMC4571992 25579192

[B15] Cai Y. Agmon N. Choi W. J. Ubide A. Stracquadanio G. Caravelli K. (2015). Intrinsic biocontainment: multiplex genome safeguards combine transcriptional and recombinational control of essential yeast genes. Proc. Natl. Acad. Sci. 112, 1803–1808. 10.1073/pnas.1424704112 25624482 PMC4330768

[B16] Calero P. Jensen S. I. Nielsen A. T. (2016). Broad-host-range ProUSER vectors enable fast characterization of inducible promoters and optimization of p-coumaric acid production in *Pseudomonas putida* KT2440. ACS Synth. Biol. 5, 741–753. 10.1021/acssynbio.6b00081 27092814

[B17] Caliando B. J. Voigt C. A. (2015). Targeted DNA degradation using a CRISPR device stably carried in the host genome. Nat. Commun. 6, 6989. 10.1038/ncomms7989 25988366 PMC4479009

[B18] Calles B. Goñi‐Moreno Á. de Lorenzo V. (2019). Digitalizing heterologous gene expression in Gram‐negative bacteria with a portable ON/OFF module. Mol. Systmens Biol. 15, e8777. 10.15252/msb.20188777 PMC6920698 31885200

[B19] Cañadas I. C. Groothuis D. Zygouropoulou M. Rodrigues R. Minton N. P. (2019). RiboCas: a universal CRISPR-based editing tool for *Clostridium* . ACS Synth. Biol. 8, 1379–1390. 10.1021/acssynbio.9b00075 31181894

[B20] Cardinale S. Joachimiak M. P. Arkin A. P. (2013). Effects of genetic variation on the *E. coli* host-circuit interface. Cell Rep. 4, 231–237. 10.1016/j.celrep.2013.06.023 23871664

[B21] Castanon O. Smith C. J. Khoshakhlagh P. Ferreira R. Güell M. Said K. (2020). CRISPR-mediated biocontainment. 10.1101/2020.02.03.922146

[B22] Chan C. T. Y. Lee J. W. Cameron D. E. Bashor C. J. Collins J. J. (2016). “Deadman” and “Passcode” microbial kill switches for bacterial containment. Nat. Chem. Biol. 12, 82–86. 10.1038/nchembio.1979 26641934 PMC4718764

[B23] Chatterjee P. Jakimo N. Jacobson J. M. (2018). Minimal PAM specificity of a highly similar SpCas9 ortholog. Sci. Adv. 4, eaau0766. 10.1126/sciadv.aau0766 30397647 PMC6200363

[B24] Chaves J. E. Wilton R. Gao Y. Munoz N. M. Burnet M. C. Schmitz Z. (2020). Evaluation of chromosomal insertion loci in the *Pseudomonas putida* KT2440 genome for predictable biosystems design. Metab. Eng. Commun. 11, e00139. 10.1016/j.mec.2020.e00139 32775199 PMC7398981

[B25] Choi K.-H. Kumar A. Schweizer H. P. (2006). A 10-min method for preparation of highly electrocompetent *Pseudomonas aeruginosa* cells: application for DNA fragment transfer between chromosomes and plasmid transformation. J. Microbiol. Methods 64, 391–397. 10.1016/j.mimet.2005.06.001 15987659

[B26] Cui L. Bikard D. (2016). Consequences of Cas9 cleavage in the chromosome of *Escherichia coli. nucleic acids research* . Nucleic Acids Res. 44, 4243–4251. 10.1093/nar/gkw223 27060147 PMC4872102

[B27] Damalas S. G. Batianis C. Martin-Pascual M. Lorenzo V. Santos V. A. P. M. (2020). SEVA 3.1: enabling interoperability of DNA assembly among the SEVA, BioBricks and Type IIS restriction enzyme standards. Microb. Biotechnol. 13, 1793–1806. 10.1111/1751-7915.13609 32710525 PMC7533339

[B28] de Lorenzo V. Krasnogor N. Schmidt M. (2021). For the sake of the bioeconomy: define what a synthetic biology chassis is. New Biotechnol. 60, 44–51. 10.1016/j.nbt.2020.08.004 32889152

[B29] Dong D. Guo M. Wang S. Zhu Y. Wang S. Xiong Z. (2017). Structural basis of CRISPR-SpyCas9 inhibition by an anti-CRISPR protein. Nature 546, 436–439. 10.1038/nature22377 28448066

[B30] Elmore J. R. Furches A. Wolff G. N. Gorday K. Guss A. M. (2017). Development of a high efficiency integration system and promoter library for rapid modification of *Pseudomonas putida* KT2440. Metab. Eng. Commun. 5, 1–8. 10.1016/j.meteno.2017.04.001 29188179 PMC5699527

[B31] Gallagher R. R. Patel J. R. Interiano A. L. Rovner A. J. Isaacs F. J. (2015). Multilayered genetic safeguards limit growth of microorganisms to defined environments. Nucleic Acids Res. 43, 1945–1954. 10.1093/nar/gku1378 25567985 PMC4330353

[B32] Gómez-García G. Ruiz-Enamorado A. Yuste L. Rojo F. Moreno R. (2021). Expression of the ISPpu9 transposase of *Pseudomonas putida* KT2440 is regulated by two small RNAs and the secondary structure of the mRNA 5′-untranslated region. Nucleic Acids Res. 49, 9211–9228. 10.1093/nar/gkab672 34379788 PMC8450116

[B33] Green A. A. Silver P. A. Collins J. J. Yin P. (2014). Toehold switches: de-novo-designed regulators of gene expression. Cell 159, 925–939. 10.1016/j.cell.2014.10.002 25417166 PMC4265554

[B34] Green R. Rogers E. J. (2013). “Transformation of chemically competent *E. coli* ,” in Methods in enzymology (Elsevier), 329–336. 10.1016/B978-0-12-418687-3.00028-8 PMC4037286 24011059

[B35] Hernández-Arranz S. Sánchez-Hevia D. Rojo F. Moreno R. (2016). Effect of Crc and Hfq proteins on the transcription, processing, and stability of the Pseudomonas putida CrcZ sRNA. RNA 22, 1902–1917. 10.1261/rna.058313.116 27777366 PMC5113210

[B36] Isaacs F. J. Dwyer D. J. Ding C. Pervouchine D. D. Cantor C. R. Collins J. J. (2004). Engineered riboregulators enable post-transcriptional control of gene expression. Nat. Biotechnol. 22, 841–847. 10.1038/nbt986 15208640

[B37] Jakočiūnas T. Jensen M. K. Keasling J. D. (2016). CRISPR/Cas9 advances engineering of microbial cell factories. Metab. Eng. 34, 44–59. 10.1016/j.ymben.2015.12.003 26707540

[B38] Jiang W. Bikard D. Cox D. Zhang F. Marraffini L. A. (2013). RNA-guided editing of bacterial genomes using CRISPR-Cas systems. Nat. Biotechnol. 31, 233–239. 10.1038/nbt.2508 23360965 PMC3748948

[B39] Jinek M. Jiang F. Taylor D. W. Sternberg S. H. Kaya E. Ma E. (2014). Structures of Cas9 endonucleases reveal RNA-mediated conformational activation. Science 343, 1247997. 10.1126/science.1247997 24505130 PMC4184034

[B40] Kim D. Lee J. W. (2020). Genetic biocontainment systems for the safe use of engineered microorganisms. Biotechnol. Bioprocess Eng. 25, 974–984. 10.1007/s12257-020-0070-1

[B41] Liu Q. Schumacher J. Wan X. Lou C. Wang B. (2018). Orthogonality and burdens of heterologous AND gate gene circuits in *E. coli* . ACS Synth. Biol. 7, 553–564. 10.1021/acssynbio.7b00328 29240998 PMC5820654

[B42] Lo T.-M. Chng S. H. Teo W. S. Cho H.-S. Chang M. W. (2016). A two-layer gene circuit for decoupling cell growth from metabolite production. Cell Syst. 3, 133–143. 10.1016/j.cels.2016.07.012 27559924

[B43] Martínez-García E. de Lorenzo V. (2017). Molecular tools and emerging strategies for deep genetic/genomic refactoring of *Pseudomonas Current Opinion in Biotechnology* . Curr. Opin. Biotechnol. 47, 120–132. 10.1016/j.copbio.2017.06.013 28738232

[B44] Martínez-García E. De Lorenzo V. (2024). *Pseudomonas putida* as a synthetic biology chassis and a metabolic engineering platform. Curr. Opin. Biotechnol. 85, 103025. 10.1016/j.copbio.2023.103025 38061264

[B45] Martin-Pascual M. Batianis C. Bruinsma L. Asin-Garcia E. Garcia-Morales L. Weusthuis R. A. (2021). A navigation guide of synthetic biology tools for Pseudomonas putida Biotechnology Advances, 107732. 10.1016/j.biotechadv.2021.107732 33785373

[B46] Mecacci S. Torregrosa-Barragán L. Asin-Garcia E. Smith R. W. (2023). Multilayered safety framework for living diagnostics in the colon. Front. Syst. Biol. 3, 1240040. 10.3389/fsysb.2023.1240040 PMC12342019 40809473

[B47] Moon T. S. Lou C. Tamsir A. Stanton B. C. Voigt C. A. (2012). Genetic programs constructed from layered logic gates in single cells. Nature 491, 249–253. 10.1038/nature11516 23041931 PMC3904217

[B48] Moreno R. Hernández-Arranz S. La Rosa R. Yuste L. Madhushani A. Shingler V. (2015). The Crc and Hfq proteins of *Pseudomonas putida* cooperate in catabolite repression and formation of ribonucleic acid complexes with specific target motifs. Environ. Microbiol. 17, 105–118. 10.1111/1462-2920.12499 24803210

[B49] Moreno R. Marzi S. Romby P. Rojo F. (2009). The Crc global regulator binds to an unpaired A-rich motif at the *Pseudomonas putida* alkS mRNA coding sequence and inhibits translation initiation. Nucleic Acids Res. 37, 7678–7690. 10.1093/nar/gkp825 19825982 PMC2794181

[B50] Moreno R. Ruiz-Manzano A. Yuste L. Rojo F. (2007). The *Pseudomonas putida* Crc global regulator is an RNA binding protein that inhibits translation of the AlkS transcriptional regulator. Mol. Microbiol. 64, 665–675. 10.1111/j.1365-2958.2007.05685.x 17462015

[B51] Nielsen A. A. K. Der B. S. Shin J. Vaidyanathan P. Paralanov V. Strychalski E. A. (2016). Genetic circuit design automation. Science 352, aac7341. 10.1126/science.aac7341 27034378

[B52] Nikel P. I. Chavarría M. Danchin A. de Lorenzo V. (2016). From dirt to industrial applications: *Pseudomonas putida* as a Synthetic Biology chassis for hosting harsh biochemical reactions. Curr. Opin. Chem. Biol. 34, 20–29. 10.1016/j.cbpa.2016.05.011 27239751

[B53] Nikel P. I. de Lorenzo V. (2018). *Pseudomonas putida* as a functional chassis for industrial biocatalysis: from native biochemistry to trans-metabolism. Metab. Eng. 50, 142–155. 10.1016/j.ymben.2018.05.005 29758287

[B54] Patinios C. Creutzburg S. C. A. Arifah A. Q. Adiego-Pérez B. Gyimah E. A. Ingham C. J. (2021). Streamlined CRISPR genome engineering in wild-type bacteria using SIBR-Cas. Nucleic Acids Res. 49, 11392–11404. 10.1093/nar/gkab893 34614191 PMC8565351

[B55] Piraner D. I. Abedi M. H. Moser B. A. Lee-Gosselin A. Shapiro M. G. (2017). Tunable thermal bioswitches for *in vivo* control of microbial therapeutics. Nat. Chem. Biol. 13, 75–80. 10.1038/nchembio.2233 27842069

[B56] Pobre V. Graça-Lopes G. Saramago M. Ankenbauer A. Takors R. Arraiano C. M. (2020). Prediction of no*vel non*-coding RNAs relevant for the growth of *Pseudomonas putida* in a bioreactor. Microbiology 166, 149–156. 10.1099/mic.0.000875 31860438

[B57] Prévost K. Salvail H. Desnoyers G. Jacques J.-F. Phaneuf É. Massé E. (2007). The small RNA RyhB activates the translation of shiA mRNA encoding a permease of shikimate, a compound involved in siderophore synthesis. Mol. Microbiol. 64, 1260–1273. 10.1111/j.1365-2958.2007.05733.x 17542919

[B58] Riglar D. T. Silver P. A. (2018). Engineering bacteria for diagnostic and therapeutic applications. Nat. Rev. Microbiol. 16, 214–225. 10.1038/nrmicro.2017.172 29398705

[B59] Rottinghaus A. G. Ferreiro A. Fishbein S. R. S. Dantas G. Moon T. S. (2022). Genetically stable CRISPR-based kill switches for engineered microbes. Nat. Commun. 13, 672. 10.1038/s41467-022-28163-5 35115506 PMC8813983

[B60] Saenger W. Orth P. Kisker C. Hillen W. Hinrichs W. (2000). The tetracycline repressor—a paradigm for a biological switch. Angew. Chem. Int. Ed. 39, 2042–2052. 10.1002/1521-3773(20000616)39:12<2042::AID-ANIE2042>3.0.CO;2-C 10941016

[B61] Siguier P. Gourbeyre E. Varani A. Ton-Hoang B. Chandler M. (2015). Everyman’s guide to bacterial insertion sequences. Microb. Spectr. 3, MDNA3–M0030. 10.1128/microbiolspec.MDNA3-0030-2014 26104715

[B62] Sonnleitner E. Bläsi U. (2014). Regulation of Hfq by the RNA CrcZ in *Pseudomonas aeruginosa* carbon catabolite repression. PLoS Genet. 10, e1004440. 10.1371/journal.pgen.1004440 24945892 PMC4063720

[B63] Sonnleitner E. Gonzalez N. Sorger‐Domenigg T. Heeb S. Richter A. S. Backofen R. (2011). The small RNA PhrS stimulates synthesis of the *Pseudomonas aeruginosa* quinolone signal. Mol. Microbiol. 80, 868–885. 10.1111/j.1365-2958.2011.07620.x 21375594

[B64] Stern M. J. Ames G. F.-L. Smith N. H. Robinson E. C. Higgins C. F. (1984). Repetitive extragenic palindromic sequences: a major component of the bacterial genome. Cell 37, 1015–1026. 10.1016/0092-8674(84)90436-7 6378385

[B65] Stirling F. Bitzan L. O’Keefe S. Redfield E. Oliver J. W. K. Way J. (2017). Rational design of evolutionarily stable microbial kill switches. Mol. Cell 68, 686–697.e3. 10.1016/j.molcel.2017.10.033 29149596 PMC5812007

[B66] Wang B. Kitney R. I. Joly N. Buck M. (2011). Engineering modular and orthogonal genetic logic gates for robust digital-like synthetic biology. Nat. Commun. 2, 508–509. 10.1038/ncomms1516 22009040 PMC3207208

[B67] Wirth N. T. Kozaeva E. Nikel P. I. (2020). Accelerated genome engineering of *Pseudomonas putida* by I‐ *Sce* I―mediated recombination and CRISPR ‐Cas9 counterselection. Microb. Biotechnol. 13, 233–249. 10.1111/1751-7915.13396 30861315 PMC6922521

[B68] Wu G. Yan Q. Jones J. A. Tang Y. J. Fong S. S. Koffas M. A. G. (2016). Metabolic burden: cornerstones in synthetic biology and metabolic engineering applications. Trends Biotechnol. 34, 652–664. 10.1016/j.tibtech.2016.02.010 26996613

[B69] Xia P.-F. Ling H. Foo J. L. Chang M. W. (2019). Synthetic genetic circuits for programmable biological functionalities. Biotechnol. Adv. 37, 107393. 10.1016/j.biotechadv.2019.04.015 31051208

[B70] Xu P. Li L. Zhang F. Stephanopoulos G. Koffas M. (2014). Improving fatty acids production by engineering dynamic pathway regulation and metabolic control. Proc. Natl. Acad. Sci. 111, 11299–11304. 10.1073/pnas.1406401111 25049420 PMC4128127

[B71] Yang H. Patel D. J. (2017). Inhibition mechanism of an anti-CRISPR suppressor AcrIIA4 targeting SpyCas9. Mol. Cell 67, 117–127.e5. 10.1016/j.molcel.2017.05.024 28602637 PMC5595222

[B72] Zadeh J. N. Steenberg C. D. Bois J. S. Wolfe B. R. Pierce M. B. Khan A. R. (2011). NUPACK: analysis and design of nucleic acid systems. J. Comput. Chem. 32, 170–173. 10.1002/jcc.21596 20645303

[B73] Zhao J. Inomata R. Kato Y. Miyagishi M. (2021). Development of aptamer-based inhibitors for CRISPR/Cas system. Nucleic Acids Res. 49, 1330–1344. 10.1093/nar/gkaa865 33123724 PMC7897479

[B74] Zobel S. Benedetti I. Eisenbach L. de Lorenzo V. Wierckx N. Blank L. M. (2015). Tn7-Based device for calibrated heterologous gene expression in *Pseudomonas putida* . ACS Synth. Biol. 4, 1341–1351. 10.1021/acssynbio.5b00058 26133359

[B75] Zuker M. (2003). Mfold web server for nucleic acid folding and hybridization prediction. Nucleic Acids Res. 31, 3406–3415. 10.1093/nar/gkg595 12824337 PMC169194

