# Supplementary material for: GenoMine: a CRISPR-Cas9-based kill switch for biocontainment of Pseudomonas putida
Source: Front Bioeng Biotechnol. 2024 Sep 16;12:1426107. doi: 10.3389/fbioe.2024.1426107 (PMC11439788; doi:10.3389/fbioe.2024.1426107)
Supplement: Supplementary file 1 [file DataSheet1.zip › Supplementary Material/GenoMine a CRISPR-Cas9-based kill-switch for biocontainment of Pseudomonas putida Supplementary Figures.docx]

**Supplementary Figures**

**GenoMine: a CRISPR-Cas9-based kill-switch for biocontainment of *Pseudomonas putida***

by

Enrique Asin-Garcia^1,2^ (https://orcid.org/0000-0001-5568-345X), Maria Martin-Pascual^1^, Claudia de Buck^1^, Max Allewijn^1^, Alexandra Müller^1^, Vitor A. P. Martins dos Santos^1, 2, 3,^ * (https://orcid.org/0000-0002-2352-9017)

^1^ Laboratory of Systems and Synthetic Biology, Wageningen University & Research, Wageningen, 6708 WE, The Netherlands

^2^ Bioprocess Engineering Group, Wageningen University & Research, Wageningen, 6700 AA, The Netherlands

^3^ LifeGlimmer GmbH, Berlin, 12163, Germany

* To whom correspondence should be addressed. Tel: +31317482865; Email: [vitor.martinsdossantos@wur.nl](mailto:vitor.martinsdossantos@wur.nl)

**Supplementary Figure S1**

Supplementary Figure S1. Cleavage survival after second exposure to test conditions of ScCas9 under the control of conventional RR12. Second exposure indicates a second cleavage survival test of cells that had survived under permissive conditions (non-induced) from the cleavage assays shown in Figure 3E. Directly re-tested cells were simply picked from the previous plate (LB-Kn), resuspended in water, and plated again onto test plates. Another test was also performed with the same cells after 24 h growth in LB-Kn. Relative cleavage survival was calculated separately for each strain in each figure as the ratio between CFU growing upon different induction conditions (purple bars represent single induction with 3MB, pink bars single induction with rhamnose and red bars double 3MB + rhamnose induction) and total CFU growing on plates without the addition of inducers (grey bars) and expressed in percentage. Only significant values are indicated for a parametric two-tailed t test between two groups, where **P* < 0.05; ***P* < 0.01; ****P* < 0.001; and *****P* < 0.0001; non-significant values were not depicted (mean ± s.d., n = 3 biological).

**Supplementary Figure S2**

Supplementary Figure S2. Effect of ScCas9 on *P. putida*’s cell populations. Percentage of cell population was calculated as the ratio between CFU obtained when transformed with either pSEVAb62-ScCas9 (targeting conditions) or pSEVAb62-dScCas9 (catalytically inactive version) and CFU obtained when transformed with an empty pSEVAb62 (non-targeting conditions) for both the GenoMine and the wild type strains (mean ± s.d., n ≥ 3 biological).
